# The Effect of Symptom-Provocation on Inhibitory Control in Obsessive-Compulsive Disorder Patients Is Contingent upon Chronotype and Time of Day

**DOI:** 10.3390/jcm12124075

**Published:** 2023-06-15

**Authors:** Omer Linkovski, Hadar Naftalovich, Mor David, Yuval Seror, Eyal Kalanthroff

**Affiliations:** 1 Department of Psychology, Bar-Ilan University, Ramat-Gan 52900, Israel; 2 Gonda Multidisciplinary Brain Research Center, Bar-Ilan University, Ramat-Gan 52900, Israel; 3 Department of Psychology, The Hebrew University of Jerusalem, Jerusalem 9190501, Israel

**Keywords:** obsessive-compulsive disorder (OCD), inhibition, stop signal task, symptom provocation, inhibitory control

## Abstract

Studies have shown that alertness can affect inhibitory control, the mechanism responsible for stopping behaviors, thoughts, or emotions. Inhibitory control is particularly important for helping individuals with Obsessive–Compulsive Disorder (OCD) resisting their symptoms. Chronotype is the mechanism governing an individual’s fluctuation of alertness throughout the day. Previous studies have shown that individuals with a ‘morning’ chronotype have worse OCD symptoms in the evening and vice versa. We administered a novel ‘symptom-provocation stop signal task’ (SP-SST), in which individually tailored OCD triggers were presented and inhibitory control was measured. Twenty-five treatment-seeking OCD patients completed the SP-SST three times per day for seven consecutive days. Stop signal reaction time (SSRT), which measures inhibitory control, was calculated separately for symptom-provocation trials and for neutral trials. Results yielded that: (a) stopping was significantly harder in the symptom-provocation compared to neutral trials, and (b) the chronotype by time-of-day interaction predicts inhibition for both symptom-provocation and neutral trials, indicating better inhibition in the optimal time of day. Furthermore, we concluded that individually tailored OCD triggers have a detrimental effect on inhibitory control. Most importantly, higher alertness levels, which can be predicted by the interaction of chronotype and time of day, affect inhibitory control, both in general and for OCD triggers specifically.

## 1. Introduction

Obsessive–compulsive disorder (OCD) is a chronic and common neuropsychiatric disorder that affects 1 in 40 people [1]. Individuals with OCD experience intrusive–repetitive thoughts (obsessions) that increase distress, which patients attempt to alleviate by engaging in ritualized or repetitive actions or mental acts (compulsions). While compulsions reduce momentary distress, they precipitate a vicious cycle linking obsessions and compulsions. In the vast majority of OCD patients, obsessions and compulsions are unwanted and patients experience their symptoms as exaggerated and/or incongruent with their goals [1]. It has been suggested that inhibitory control plays a crucial part in the ability or inability to stop compulsive behaviors [2,3,4,5,6,7]. Different processes affecting inhibitory control may mediate patients’ performance and thus, also influence our clinical and scientific understanding of OCD. One such process is alertness levels, which have been shown to effect inhibition significantly. Therefore, the current study assesses how circadian alertness changes are associated with altered inhibitory control and help us predict when our innate abilities to resist unwanted behaviors, thoughts, or emotions may be facilitated or impaired.

**Figure 1:**
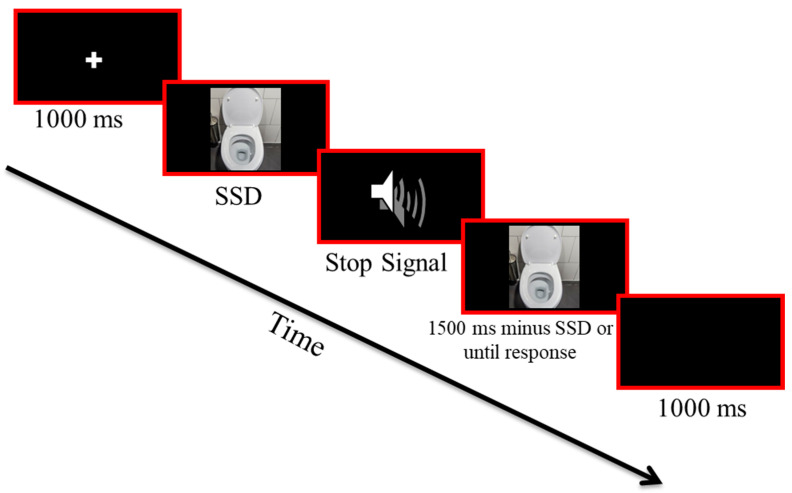
The symptom-provocation stop signal task (SP-SST). Example of a symptom-provocation stop signal trial. Each trial started with a 1000 ms fixation (a white plus sign at the center of a black screen). Following this, a visual go stimulus appeared (i.e., a symptom-provocation picture or a neutral picture) appeared randomly. The go stimulus stayed in view for 1500 ms or until a key press. In stop signal trials, an auditory tone was presented shortly after the appearance of the go-signal. The duration between the go and stop signal (SSD) was subjected to a tracking procedure that was applied separately for each condition (symptom provocation vs. neutral). Each trial ended with a 1000 ms inter-trials interval (black screen).

There is an ongoing debate regarding the role of inhibitory control in the onset and maintenance of OCD. Inhibitory control is a mechanism that underlies our ability to inhibit behaviors, thoughts, and emotions [8,9,10]. The stop signal task is a validated measure for inhibitory control [11]. In the classic task, participants are asked to respond as quickly and accurately as possible to a go signal (e.g., a circle or square) while also asked to refrain from any reaction when a stop signal (e.g., auditory beep) is introduced. Via a tracking procedure which manipulates the duration between the go and stop signals (see Section 2 Methods and Figure 1), the task allows us to calculate the stop signal reaction time (SSRT), which is a reliable and valid measure for inhibitory control. While some contend that inhibitory control deficits are central to the etiology of OCD [12], others argue that OCD patients’ inhibitory control changes are a result of their symptoms [13] or medication status [14]. Regardless of this debate on the role of inhibition in the etiology of the disorder, it is clear that inhibitory control is required to inhibit any unwanted behaviors, thoughts or emotions [15,16,17,18,19,20] and thus, for overcoming OCD symptoms. Therefore, it is crucial to understand which factors can either facilitate or impede inhibitory control to maximize our chances of successfully overcoming unwanted behaviors, thoughts, or emotions.

One such factor may be alertness. Alertness is a bottom-up attentional system which is mediated by levels of norepinephrine released from the locus coeruleus. Posner and Petersen suggested that alertness, spatial orienting, and executive functioning or inhibitory control are key attentional networks [21,22]. They predicted that fluctuations in alertness will alter the efficiency of inhibitory control and spatial orienting of attention. This cognitive hypothesis regarding the interaction between alertness and inhibitory control has been validated by neurochemical and anatomical findings. Norepinephrine regulates wakefulness [23], possibly via increasing sensitivity to external cues [24]. Aston-Jones’ seminal work showed a direct link between the locus coeruleus, which generates norepinephrine, and the right inferior frontal gyrus, a brain region that is highly involved in inhibitory control [25,26]. Furthermore, increasing norepinephrine with pharmacotherapy has also been shown to improve inhibitory control [27,28]. These findings provide strong neurological evidence that alertness and inhibition regularly interact. Even behaviorally, increasing healthy adults’ alertness has been shown to improve inhibitory control [29]. In OCD patients, alertness, through its effect on inhibitory control, may contribute to the ability to resist symptoms [30,31]. Naftalovich and colleagues demonstrated that alertness levels, manipulated using caffeine, can attenuate the ability to resist compulsive urges, potentially through the effect of alertness on inhibition [32]. Similarly, preliminary evidence indicates that light therapy, in which patients are exposed to bright light during morning hours to regulate circadian rhythms, is associated with improved OCD treatment outcomes and alleviates OCD severity [33]. Importantly, alertness levels are determined by various factors, including circadian rhythm. Therefore, one way to test the links between alertness and inhibitory control in OCD patients is to assess inhibitory control under different alertness levels. There are various methods through which one can increase alertness, though one especially promising and ecological method of doing so is through estimating alertness levels based on chronobiology.

Alertness is tightly linked to chronobiology and circadian rhythm [34]. Circadian rhythm is a daily cycle involving major physiological and neural processes spanning wakefulness, endocrine regulation, the immune system, and cognitive abilities [35,36]. Importantly, individuals differ in the patterns of circadian rhythms. One way to quantify circadian differences is to assess one’s chronotype-optimal time of day for different activities and mental alertness [37,38]. Individuals with strong morningness tendencies (‘morning types’) exhibit optimal alertness during the morning compared to the evening and individuals with strong eveningness tendencies (‘evening types’) experience optimal alertness during the evening [37]. Indeed, chronotype has been shown to affect various physiological and psychological mechanisms [39,40,41], and specifically, chronotype affects the fluctuations of alertness levels throughout the day [34,38,42] including modulated locus coeruleus activity [43]. Thus, based on the interaction between chronotype and time of day on alertness, the known effect of alertness on inhibitory control, and the role of inhibitory control in resisting OCD symptoms, it may be possible to predict when OCD symptoms may be easier or more difficult to resist based on the knowledge of one’s optimal time of day.

Despite the evidence supporting the effect of alertness on OCD symptoms [44,45,46], only one study directly assessed OCD symptom fluctuations as a function of chronotype and time of day. Naftalovich et al. monitored OCD patients for 7 days and showed that patients’ symptom fluctuations can be explained as a function of chronotype and time of day [45]. These researchers were able to demonstrate that OCD patients who are morning types exhibited more symptoms in the evening whilst OCD patients who are evening types exhibited more symptoms in the morning. In other words, OCD symptoms were more frequent and more severe in patients’ non-optimal time [44,45,46]. Although this effect was hypothesized to be related to inhibitory control levels, inhibition was not directly tested in that study. Taken together with the information provided above, these findings provide preliminary support for the hypothesis that OCD symptom severity throughout the day can be predicted based on knowledge of one’s optimal time of day and their own individual chronotype. In other words, this provides initial evidence that individuals with OCD will have an easier time inhibiting symptoms in their optimal time of day (e.g., morning for *morning* types) as opposed to their non-optimal time of day (e.g., morning for *evening* types).

The goal of the current study was to assess the effect of alertness on the ability of OCD patients to execute inhibition when their OCD symptoms are provoked. Therefore, we conducted a pilot study with a small sample of treatment-seeking OCD patients in which inhibitory control was measured by the novel symptom-provocation stop signal task (SP-SST) at different times of day, in both morning- and evening-type OCD patients. This task enabled us to measure inhibitory control in response to both neutral images and obsession-inducing images. We hypothesized that: (a) OCD patients will exhibit worse inhibitory control in response to symptom-provoking images, compared to neutral images, and that (b) OCD patients will exhibit reduced inhibition in general, and more so to the symptom-provocation trials, in their non-optimal time of day based on their chronotype.

## 2. Methods

### 2.1. Study Design

This study was conducted as part of larger clinical study at The Hebrew University of Jerusalem and approved by the institutional review board (HUJI-23012023). After providing written informed consent, patients completed baseline clinical assessments and self-report questionnaires. Next, the task was installed on the patients’ personal computers (desktops or laptops) and the patients completed the symptom-provocation stop signal task (SP-SST) from home, three times a day for seven consecutive days.

### 2.2. Participants

Twenty-five adults between the ages of 18 and 45 were recruited for a treatment study through the use of advertisements, word of mouth, and clinical referrals. To be eligible, individuals had to have a primary diagnosis of OCD (duration of ≥1 year), and a Yale–Brown Obsessive Compulsive Scale (Y-BOCS; score ≥ 16) [47]. Table 1 describes the full demographic and clinical characteristics of the sample. Participants who leaned toward ‘morning-types’ (MEQ ≥ 45, *N* = 10) or leaned toward ‘evening-types’ (MEQ < 45; *N* = 15) took part in the current study (see description below of the MEQ). Exclusion criteria included manic episode (current or past), psychosis (current or past), current prominent suicidal ideation, substance abuse or dependence in the past 6 months, current severe major depressive disorder, or any neurological disorder. Patients were not excluded if they had other comorbid disorders, as long as OCD symptoms were the most severe and impairing of the pertinent diagnoses. Patients on psychiatric medication were eligible if they were on a stable dose for at least six weeks prior to study entry and remained on the same dose for the duration of the study. Eligibility was determined by skilled PhD-level licensed clinical psychologists using the Structured Clinical Interview for DSM-V (SCID-5) [48]. Finally, all participants completed at least 20 (out of 21) SP-SST sessions.

### 2.3. Clinical Assessments

Diagnoses and clinical assessments were conducted by one of two trained independent evaluators with expertise in OCD who had no other contact with study patients. Independent evaluators conducted weekly reliability training. After a brief phone screening (conducted by a research assistant), potential participants were invited to the lab and diagnoses were done to confirm eligibility, using the SCID-5 [48] and the Y-BOCS—a structured interview for OCD that has excellent psychometric properties [47]. In addition, patients completed two self-report questionnaires: The Obsessive–Compulsive Inventory—Revised (OCI-R), an 18-item self-report scale that assesses the distress associated with obsessions and compulsions; and the Beck Depression Inventory—II (BDI-II), a 21-item self-report scale that assesses the severity of depressive symptoms. Both the OCI-R and BDI-II have strong psychometric properties [49,50,51,52].

### 2.4. The Symptom-Provocation Stop Signal Task (SP-SST)

The SP-SST builds on the generic stop signal task. In the generic stop signal task, participants make quick responses to stimuli, such as indicating whether a shape is a circle or a square. In a minority of trials, after participants see the target stimulus, a stop signal is introduced, indicating they should inhibit their response. The task modifies the delay between the go and stop signals based on the participant’s performance to eventually reveal the time it takes participants to stop, i.e., the stop signal reaction time (SSRT). In the current study, SSRT was calculated using the integration method following the recent guidelines outlined in the consensus statement (see Analysis section; [11]).

The SP-SST (Figure 1) was modified from the generic stop signal task using OpenSesame 3.0 (using Python 2.7.6). Each session included 400 experimental trials (100 of which were stop trials). In each trial, a picture was presented on the computer screen and patients were asked to respond as quickly and as accurately as possible to the background color of the picture (light vs. dark) by pressing the ‘z’ or ‘m’ keys on the computer keyboard. In 50% of the trials, personalized OCD-provoking pictures were presented (e.g., a picture of a skin infection for a patient with specific skin-related contamination intrusions), whereas the other 50% of the trials were neutral trials (e.g., flowers, houses). In a random selection of 25% of the trials, a stop signal (i.e., an auditory tone, 750 Hz, 75 ms) appeared shortly after the visual stimuli appeared, signaling that the response (pressing a button on the keyboard) should be inhibited. The stop signal was presented after a variable stop signal delay (SSD) that was initially set at 250 ms and was continuously adjusted according to separate staircase tracking procedures that were applied for each condition separately (symptom-provoking vs. natural) to obtain a probability of stopping of 50% for each condition. After each successful stop (following a stop signal) the SSD (for the same condition) was extended by 20 ms and after each unsuccessful stopping, the SSD was shortened by 20 ms. Participants were asked to discriminate between light and dark pictures and the instructions indicated that they should press the left/right keys with the corresponding index finger, as quickly and accurately as possible. The researcher emphasized not to wait for a potential stop signal (i.e., to avoid developing a waiting strategy). Reaction time (RT) was calculated from the appearance of the go stimulus to the response. Trial order was randomized. Participants were asked to complete three sessions of the task every day for seven consecutive days, and the researchers emphasized that the participants should leave at least four hours between two sessions.

To personalize the task, the symptom-provoking pictures were selected by the evaluator and patient together at the end of the clinical assessment session and after completing the Y-BCOS structured interview [47]. Stimuli were chosen to effectively trigger the urge to do a compulsion without causing overwhelming anxiety. Images were obtained through different online resources (e.g., Google image search). Patients were asked to send the results files (generated by the program) to the research assistant by email at the end of every day.

### 2.5. The Morningness–Eveningness Questionnaire (MEQ)

The MEQ measures peak alertness patterns based on one’s chronotype. It includes 19 multiple-choice, 4-point scale questions. The sum provides a score ranging from 16 to 86; lower scores represent “evening types” and higher scores represent “morning types”. For the goal of the current study, we used 45 as the cutoff point. Internal consistency for this questionnaire is a Cronbach’s alpha of 0.77–0.80 [53,54].

### 2.6. Statistical Analysis

All statistical analyses were conducted using IBM SPSS 25. First, stop signal reaction time (SSRT) was calculated for each participant in each condition separately, using the integration method [11,55]. For each participant in each condition separately, we extracted the *n*th RT from the RT distribution or correct no-stop trials. *n* is the number of correct no-stop signal trials × *p* (response|signal). SSRT was then calculated as the *n*th RT-mean SSD (which was adjusted for each participant in each condition separately). Each SP-SST session was considered a ‘morning session’ if participants completed the session before 11 a.m., a ‘mid-day session’ if participants completed the session between 11 a.m. and 5 p.m., or an ‘evening session’ if participants completed the session after 5 p.m. Next, SSRT data were subjected to a three-way mixed model analysis of variance (ANOVA) with valence condition (symptom-provocation vs. neutral) and time of day (morning vs. evening) as within-subject factors and chronotype group (morning type vs. evening type) as a between-subject factor.

Next, a General Estimating Equation (GEE) with an autoregressive correlation of order 1 (AR1; [56]) was carried out on SSRT for the symptom provocation condition (for this analysis, we did not use the SSRT for the neutral condition) at the two time points (morning and evening) with subject entered as a random factor, and the interaction between chronotype (entered as a covariate) and time of day (entered as a factor) as a predictor. GEE is a method for modeling longitudinal or clustered data by estimating the parameters of a generalized linear model with a possible unmeasured correlation between observations from different timepoints. The GEE (AR1) analysis was conducted to control for the presence of autocorrelations between time points and was chosen because it addresses how past time points may influence future time points.

## 3. Results

**Figure 2:**
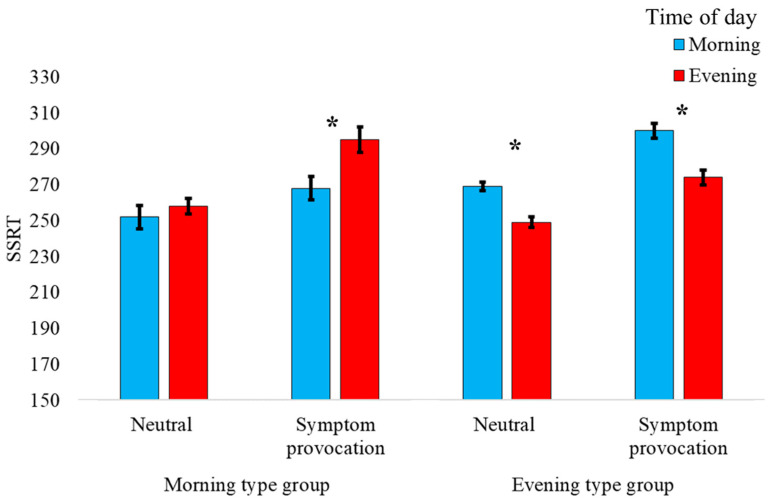
Mean stop signal reaction time (SSRT) for the symptom provocation stop signal task (SP-SST) as a function of time of day and chronotype group. Time of day represents measurement time. Error bars represent one standard error from the mean. *—significant at *p* < 0.05 level.

Consistent with our first hypothesis, that inhibition to the symptom-provocation condition will be reduced compared to the neutral condition, we found a significant main effect for the valence condition, *F*_(1, 23)_ = 23.628, *p* < 0.001, $\eta_{p}^{2}$ = 0.507, indicating that the symptom-provocation stimuli had a detrimental effect on inhibitory control (Table 2). Consistent with our hypothesis that the ability to stop is affected by the interaction between time of day and chronotype, there was a significant two-way interaction between time of day and chronotype group, *F*_(1, 23)_ = 6.533, *p* = 0.018, $\eta_{p}^{2}$ = 0.221, indicating shorter SSRTs (better inhibition) in the morning compared to the evening, for the ‘morning-type’ group and longer SSRT (worst inhibition) in the morning compared to the evening for the ‘evening-type’ group (Table 2; Figure 2). However, counter to our hypothesis, the three-way interaction between valence condition, time of day, and chronotype group was not significant, *F*_(1, 23)_ = 1.918, *p* = 0.179, $\eta_{p}^{2}$ = 0.077, indicating that the effect of chronotype and time of day on SSRT was not modulated by the valence condition (Figure 2).

Consistent with the previous analysis, the GEE (AR1) analysis yielded a significant time of day X chronotype group interaction (Wald Chi-Square = 5.165, *p* = 0.023), indicating that chronotype alignment significantly predicted SSRT at different timepoints throughout the day. Notably, neither time of day (Wald Chi-Square = 0.002, *p* = 0.969) nor chronotype group (Wald Chi-Square = 0.366, *p* = 0.545) alone significantly predicted SSRT at the different timepoints, indicating that SSRT is not generally improved at different times of the day, nor is it deficient for specific chronotypes, but rather, it is only the interaction between the two which predicts within-day fluctuations in SSRT.

## 4. Discussion

The current study assessed inhibitory control to neutral vs. symptom-provoking images, in a sample of treatment seeking OCD patients as a function of chronotype and time of day. We hypothesized that when OCD patients encounter symptom-provoking stimuli, their inhibitory control will be impaired compared to when they encounter neutral stimuli. Furthermore, we hypothesized that OCD patients’ inhibitory control would be impaired during non-optimal times of day based on their chronotype, and mostly for the symptom provocation condition. As predicted, patients exhibited less efficient inhibitory control in response to symptom-provoking stimuli compared to neutral stimuli. Most importantly, inhibitory control was significantly affected by the interaction between chronotype and time of day—patients had more efficient inhibitory control when their alertness was optimal per their chronotype. Interestingly, we were not able to show that this interaction affects neutral and symptom-provoking stimuli in a different way. These findings provide initial support that inhibition may be influenced by situational factors, such as alertness levels, and that the impairment in inhibition may be individually predicted by examining the interaction between chronotype and time of day. That inhibition to type of image was not affected by the chronotype and time of day interaction suggests that a broader mechanism may be at play.

Our first finding, demonstrating less efficient inhibitory control for symptom-provocation, lends support to the notion that inhibition is affected by the symptoms in individuals with OCD. This finding is consistent with previous findings in the field. Adams [57] investigated an analogue sample of participants with high and low contamination fears and found that disorder-relevant or emotionally arousing trials led to more difficulty in inhibiting actions. In a similar vein, Hudiburgh and colleagues reported a correlation between OCD symptoms and increased emotional impulsivity, which may reflect less efficient inhibitory control [58]. These findings on the relationship between symptom-provocation and inhibitory control are further supported by functional imaging studies, which shed light on the neural mechanism underlying this effect of symptom-provocation on inhibitory control in OCD patients. OCD patients experience increased amygdala activation and enhanced connectivity between the amygdala and the prefrontal cortex during symptom provocation [59,60]. As broad regions of the prefrontal cortex mediate inhibitory control, increased functional connectivity with the amygdala may underlie less efficient inhibitory control. Consistent with these neuroimaging findings, the relationship between symptom-provoking images and less efficient inhibitory control may be explained by the fact that the symptom-provocation images induce negative emotion and several investigations have demonstrated the detrimental effect of negative emotion on inhibition [10,18]. Indeed, a recent model of OCD suggested that the reduction in inhibition caused by the negative affect of anxiety (which is provoked by the OCD symptoms) plays a crucial role in the vicious cycle of OCD, as inhibition is highly needed to stop the compulsive behaviors triggered by the very same anxiety [61]. It is also possible that symptom-provoking stimuli induce obsessions that overload OCD patients’ inhibition and limit patients’ ability to inhibit their responses [13,62]. On a neurobiological level, patients’ reduced ability to inhibit responses to symptom-provoking stimuli in external tasks (like the stop signal task) may reflect interference in downregulating their default mode network—a large-scale brain network associated with self-referential processes [63,64,65]. Taken together, the above studies combined with the findings of the current study provide support for the notion that symptom-provocation is associated with impaired inhibition in individuals with OCD, a mechanism which may underlie the vicious cycle of obsessions/anxiety and compulsions in OCD.

The detrimental effect of symptom-provocation images on inhibitory control might also be discussed in terms of increased action tendencies. It has been shown that emotions, such as those triggered by the symptom-provocation images, might also elicit pre-potent responses or action tendencies [66,67]. For example, feeling glee is associated with approach behaviors and feeling distress is associated with moving away from a stimulus [68]. In the current study, negative emotions, triggered by the stimuli, might have increased action tendencies, which in turn had a detrimental effect on inhibition. This resembles the process in which individuals with OCD engage in repetitive acts—compulsions—when faced with stimuli that provoke their symptoms. The fact that symptom-provoking images reduced inhibitory control might suggest that these stimuli are associated with extremely potent action tendencies, which are difficult to inhibit [69].

The main finding of the current study indicates that chronotype affects inhibitory control as a function of time of day. This finding suggests that individuals with OCD who are morning types, and whose alertness is optimal in the morning, will have more efficient inhibitory control in the morning compared to the evening. Similarly, individuals with OCD with superior alertness in the evening will exert more efficient inhibitory control in the evening. This finding corresponds with the recent finding of Naftalovich and colleagues described above, which found that OCD patients reported lower (i.e., better) symptom severity during their optimal time of day based on their chronotype [45]. This study was conducted as a daily monitoring study in which patients completed questionnaires regarding their chronotype and clinical symptoms then reported on the severity of their OCD symptoms daily for seven days. Taken together, the finding that inhibitory control was more efficient during optimal times of day, based on chronotype, suggests that we can predict symptom severity fluctuations in OCD based on the interaction between chronotype and time of day. Importantly, the current study adds to the extant literature by showing that the mechanism behind this relationship may be the attenuation of inhibitory control by the natural fluctuations of alertness based on one’s chronotype. Interestingly, in our sample, the effect of chronotype alignment was not modulated by stimulus valence. This surprising finding suggests that as chronotype alignment reflects optimal alertness levels, and alertness increases inhibitory control [29], the effect of chronotype alignment on OCD symptoms reflects a global effect on inhibition in general, and not necessarily on inhibition to OCD symptoms specifically. This finding also suggests that the effect of chronotype alignment on inhibitory control should be replicated in individuals with other psychopathologies. Although this suggestion awaits future investigation, some indications do exist as to the effect of chronotype alignment on inhibitory control in non-clinical samples [70,71,72].

Importantly, there are two possible implications of the current findings regarding clinical practice. First, we provide a more nuanced view of chronotype in OCD. We suggest that it is useful to consider chronotype-alignment during treatment planning. It is important for both patients and clinicians to be aware of times of day which might be ‘riskier’ for them in terms of stumbles in response prevention. This information can then be used in scheduling treatment sessions and homework. For example, if the patient has an extreme morning chronotype, the clinician may assign more intense exposures during the morning hours and then move to exposures during the evening, since the patient’s inhibitory control will be less efficient at that time and it will be more challenging for them to refrain from engaging in compulsions. Second, clinicians may also use our findings to enhance psychoeducation for OCD patients during exposure and response-prevention treatment. For example, our results demonstrated that patients struggle to inhibit even simple responses once they see a stimulus which provokes symptoms. Therefore, it may not be solely distress which triggers symptoms, but rather, inefficient inhibition which may impair one’s ability to cope with distressing stimuli, such that perhaps the natural fluctuations in inhibitory control makes stopping harder during non-optimal times and potentially makes unsuccessful attempts at inhibiting symptom-provoking stimuli more distressing.

As a preliminary study, a few limitations of the current study should be noted. First, our findings warrant replication in larger samples with varying levels of symptom severity. In addition, given that the current study tested individuals who ‘lean’ toward morning or evening type, our findings should also be replicated on ‘definite’ morning and evening type individuals. Second, our experimental task included neutral and symptom-provoking stimuli. While inhibitory control was tracked separately for each stimulus type, the symptom-provocation stimuli might have affected participants throughout the task. A third limitation relates to the ecological nature of the study. Participants completed the task in the morning and in the evening, but actual completion time varied within these segments of the day between patients and within patients over the week. It is possible that participants’ chronotype affected completion times and reduced the magnitude of misalignment between chronotype and task-completion time. Future studies may include constant completion times of the experimental task—this suggested design may be challenging for patients and so researchers will need to plan for increasing patients’ motivation and adherence. Future brain imaging studies may assess OCD patients’ activity of locus coeruleus in the morning and evening as a more direct measure of alertness.

## 5. Conclusions

To conclude, the current study showed that certain stimuli or situational factors can influence whether we will successfully overcome unwanted behaviors, thoughts, or emotions [73]. First, we showed that when individuals with OCD encounter symptom-provoking stimuli, their inhibitory control becomes less efficient than when they encounter neutral stimuli. This finding supports the growing literature indicating that negative affect impairs inhibitory control. Exactly which factors impair inhibitory control and how this effect can be mitigated requires further study. Finally, the current study also shows that independent of stimuli type, non-optimal time of day, based on one’s chronotype, may impair inhibitory control and affect our ability to overcome unwanted behaviors, thoughts, and emotions. We suggest that the mechanism behind this is as such: firstly, our chronotype influences the natural fluctuations of alertness such that certain times of day will be more or less optimal based on the different chronotypes; next, these fluctuations influence when inhibitory control will be more or less optimal, and as a result, will mediate relative success in overcoming unwanted behaviors, thoughts, or emotions.

## Figures and Tables

**Table 1:** Demographics and Clinical Characteristics.

	Morning Type Group (*N* = 10)		Evening Type Group (*N* = 15)	
**Demographics**				
Age (years; mean, SD)	35.3	(13.6)	27.7	(9.6)
Education (years; mean, SD)	14.5	(2.9)	13.5	(1.5)
Sex (n, % female/male)	3/7	(30/70%)	5/10	(33/66%)
Current SRI (n, % on SRI)	4	(40%)	9	(60%)
**Clinical Characteristics**				
Y-BOCS Total (mean, SD)	28.6	(5.5)	30.8	(3.6)
MEQ	50.3	(3.7)	36	(6.6)
OCI-R (mean, SD)	29.8	(11.8)	31.7	(14.4)
BDI-II	20.1	(10.2)	21.4	(11.9)
Current comorbid disorders (N)	Depression (4), Dysthymia (1), ADHD (2), Social anxiety (2), GAD (1), Specific phobia (3), BDD (1), EA (1).		Depression (6), Dysthymia (2), ADHD (4), Social anxiety (2), GAD (3), Specific phobia (5), BDD (3).	

SRI = serotonin reuptake inhibitors; Y-BOCS = Yale–Brown Obsessive Compulsive Scale; MEQ = morningness–eveningness Questionnaire; OCI-R = obsessive–compulsive inventory—revised; BDI-II = Beck Depression inventory—II; SD = standard deviation; ADHD = attention deficit/hyperactively disorder; GAD = generalized anxiety disorder; BDD = body dysmorphic disorder; EA = eating disorder.

**Table 2:** Results.

		Morning Type Group (*N* = 10)		Evening Type Group (*N* = 15)	
Session		Morning	Evening	Morning	Evening
nsRT	Neutral	564 (18)	574 (30)	594 (10)	564 (21)
	Symptom provocation	600 (13)	615 (12)	596 (10)	586 (15)
SSRT	Neutral	252 (13)	258 (9)	269 (5)	249 (6)
	Symptom provocation	268 (13)	295 (14)	300 (8)	274 (8)
*p*(r|s)	Neutral	0.52	0.55	0.53	0.56
	Symptom provocation	0.53	0.55	0.51	0.53

Note. Reaction time in milliseconds (one standard error of the mean); nsRT = reaction time of correct responses for no-stop trials; SSRT = stop signal reaction time; *p*(r|s) = proportion of erroneous responses to stop signal trials.

## Data Availability

The data presented in this study are available on request from the corresponding author. The data are not publicly available due to privacy restrictions.
